# Formative Usability Evaluation of a Three-Way Digital Healthcare System for the People with Disabilities and Their Caregivers: A Cross-Sectional Study

**DOI:** 10.3390/healthcare10112325

**Published:** 2022-11-20

**Authors:** Ju Hee Kim, Young-Hyeon Bae, Sung Shin Kim, Minyoung Lee, Seung Hee Ho

**Affiliations:** 1 Department of Healthcare and Public Health, Rehabilitation Research Institute, Korea National Rehabilitation Center, Seoul 01022, Republic of Korea; 2 Department of Clinical Rehabilitation Research, Rehabilitation Research Institute, Korea National Rehabilitation Center, Seoul 01022, Republic of Korea

**Keywords:** people with disabilities, caregivers, usability evaluation, three-way digital healthcare system, formative

## Abstract

During the COVID-19 pandemic, there was a growing awareness about the importance of building a health and safety net based on digital healthcare systems, such as ICT-based local community online services and patient monitoring technology. This study was conducted with the aim of evaluating the formative usability of a three-way digital healthcare system, which had been developed to build a health and safety net for people with disabilities and deriving the directions for system improvement in order for them to be used as basic data for further system enhancement. A formative usability evaluation of a three-way digital healthcare system was performed with the participation of 43 healthcare professionals, using the 10-item System Usability Scale (SUS) and five items for satisfaction evaluation. Each item was rated on a five-point Likert scale, with the result converted to a scale of 100. Analysis was performed using the average score and the acceptable system usability level. The overall mean SUS score was 62.4, which corresponds to Grade D according to the SUS grading scale, and the below-average items were complexity (Q2), convenience (Q8), simplicity (Q3), professionalism (technician support, prior learning) (Q4, Q10), and learnability (Q7). The overall mean user satisfaction was 71.2 points, where overall satisfaction, system architecture and understandability, and continuous use intention were marked with below-average scores. The SUS D grade is interpreted as “fair” and the water solubility is “almost acceptable”. For the usability enhancement of the newly developed a three-way digital healthcare system, the overall direction for system architecture improvement was analyzed centering on complexity (Q2), convenience (Q8), professionalism (technician support, prior learning) (Q4, Q10), learnability (Q7), and simplicity (Q3). Efforts need to be directed at enhancing system satisfaction and continuance rate by deriving detailed system improvement strategies and achieving system enhancement to reflect the opinions of not only experts but also users.

## 1. Introduction

The challenges posed by the COVID-19 pandemic situation, notably to people with disabilities, include incidence and deterioration of health problems, exposure to lifestyle-related diseases (diabetes, hypertension, obesity), and restrictions on healthcare utilization and information acquisition, which necessitates a wide range of supports with regard to healthcare utilization, mental health, transportation, and personal care [1]. Long-term isolation due to infection prevention measures, such as self-quarantine and social distancing, can exacerbate existing health problems or create new ones [2]. In addition, restrictions on daily living due to the sudden discontinuation of mobility support can cause great confusion and fear in people with disabilities who are in need of mobility support [3].

In order to support the health, safety, quality of life, and daily life of people with disabilities, various services such as non-face-to-face services using information and communication technology (ICT) and PC or mobile health (mHealth) based on patient monitoring technology a digital healthcare system is being developed and utilized [4,5].

The iMHere system, for example, developed by the University of Pittsburgh, USA, is a three-way (patient–caregiver–physician) linkage system for patients with chronic diseases and disabilities. This system allows efficient health management, increased drug compliance, and smooth communication, preventing the development of further complications. The iMHere system improves the quality of life of patients [6].

Similarly, the ePRO system, developed by the University of Toronto, Canada, is a three-way linkage system for patients with chronic diseases and disabilities. This system allows patients, caregivers, and healthcare professionals to utilize the My Goal Tracker (which encompasses one’s physical health, mental and cognitive health, activity, pain, and diet) and the Hospital CheckOut program [7].

Digital healthcare technology is emerging as a potentially low-cost scalable platform that decreases the burden on caregivers [8]. Caregivers of people with disabilities suffer from great psychological and physiological stress [9]. Although the health status of caregivers is an essential factor in the burden of care [10], healthcare services for caregivers are often insufficient.

Currently, there is no health management service that records the health information of both people with disabilities and their caregivers, even with the use of information and communication technology. Since the key element of health promotion behavior among people with disabilities is family support, a three-way sharing-based digital healthcare system for people with disabilities and their caregivers should be established. This would allow access to good physical and psychological health among people with disabilities and their caregivers.

Therefore, considering that the main factor in the health promotion behavior of the disabled is family support [11], it is necessary to establish a health and safety net by maintaining accessibility between the disabled and their guardians and providing physical and psychological stability.

A previous study examined, during the COVID-19 pandemic, changes in the health status and healthcare routine of the disabled and caregivers, as well as changes in caregiving, daily life, social relationships, and usage of services and facilities, acquirement of information on responses to infectious diseases, and the requirement of non-face-to-face services. Additionally, we confirmed the need for the organic linkage of community and public resources in times of disaster. We also established the urgency of preparing specific and detailed support measures customized for various subjects who belong to vulnerable social groups [12].

**Figure 1:**
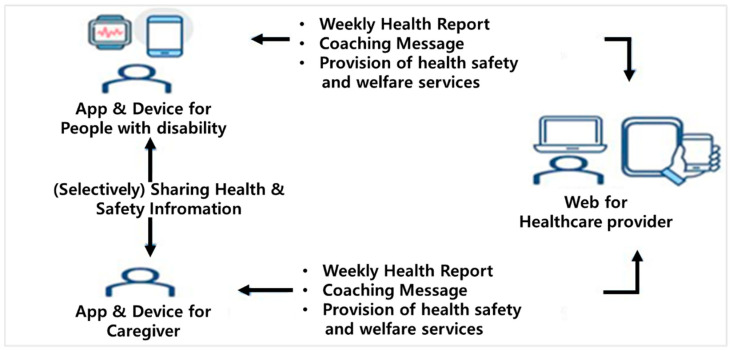
A Three-Way Digital Healthcare System for the People with Disabilities and Their Caregivers.

Recently, a prototype three-way digital healthcare system for people with disabilities and their caregivers has been developed to support the safety and health management of people with disabilities and their caregiver’s in the COVID-19 pandemic and to respond quickly in crisis situations (Figure 1).

The usability evaluation includes a formative usability evaluation, conducted in the middle part, and a general usability evaluation, conducted in the latter part according to its time and purpose [13]. Therefore, it is necessary to conduct a formative usability evaluation to confirm the feasibility of the developed prototype digital healthcare system.

In this study, with the participation of healthcare professionals, a formative usability evaluation was performed on a three-way digital healthcare system for people with disabilities and their caregivers with the aim of deriving the directions for system improvement in order for them to be used as basic data for system enhancement in the future.

## 2. Materials and Methods

### 2.1. Participants

After the first development of a Three-Way Digital Healthcare System, a usability evaluation was conducted targeting healthcare professionals to identify problems through the first formative evaluation. Forty-three healthcare professionals related to people with disabilities (experts in three fields: academia, public health and welfare, and industry) were randomly selected from the Seoul metropolitan area based on convenience sampling, and a questionnaire survey was conducted for usability evaluation of the newly developed a three-way digital healthcare system for the people with disabilities and their caregivers.

After understanding the purpose of the study via the explanations given in the questionnaire, the participants provided their consent to participate in the study. The questionnaire included explanations about the main features of the digital healthcare system based on the health and safety net for people with disabilities, system architecture, and user interface (UI), a 15-item questionnaire consisting of the 10-item System Usability Scale (SUS), 5 items for user satisfaction, and an open-ended expert questionnaire. The completed questionnaires were retrieved online for analysis.

### 2.2. A Three-Way Digital Healthcare System for the People with Disabilities and Their Caregivers

Since the key element of health promotion behavior among people with disabilities is family support, a three-way sharing-based digital healthcare system for people with disabilities and their caregivers was established by healthcare professionals. People with disabilities and their caregivers receive health status monitoring and appropriate health management services through a Three-Way Digital Healthcare System. At this time, the people with disabilities and the caregiver could mutually know each other’s health status information (optionally). Through this system, healthcare professionals could identify the health conditions of both the disabled and caregivers and provide appropriate coaching and health, safety, and welfare services (Figure 1). 

A three-way, sharing-based digital healthcare system for people with disabilities and their caregivers was established in four steps. STEP 1. The desired mobile application requirements were collected and analyzed. -> STEP 2. Designing the daily health mobile application (assessment, prioritization of needs, and UI drawing). -> STEP 3. Implementation of the mobile application. -> STEP 4. Testing of the mobile application [14].

The main features: (1) providing customized health management content for people with disabilities; (2) encouraging healthy life of people with disabilities and their caregivers by sharing information on health and healthy life among people with disabilities, caregivers, and healthcare professionals; (3) providing information on health, safety, and welfare; and (4) identifying health and safety needs at any given time. The system consists of six sections and a user interface (UI).

**Figure 2:**
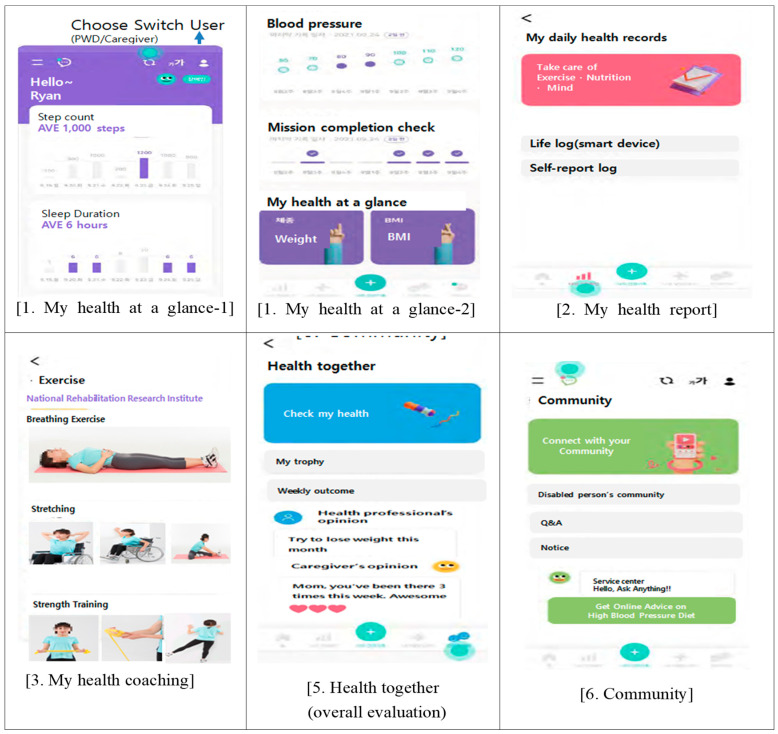
UIs of Three-Way Digital Healthcare System for the People with Disabilities and Their Caregivers.

The 6 sections of the digital healthcare system are: (1) My health at a glance, (2) My profile, (3) My health, (4) My wellbeing assistant, (5) Health together, and (6) Communities. Sub-sections are outlined in Table 1. Figure 2 illustrates the UIs developed for user participation based on the system architecture in Table 1.

### 2.3. Evaluation Instruments

SUS is a questionnaire that can be used for evaluating the usability of various products and services. SUS is a practical and reliable tool for measuring perceived simplicity by providing a rapid and efficient means to assess the usability of a product or design. It can be applied to a wide range of digital products and services to help user experience (UX) professionals determine whether a design solution has any usability problems [15]. 

Each item was rated on a 5-point Likert scale (1 = strongly disagree, 5 = strongly agree). Of the 10 assessment items, odd-number items are positive questions, where a higher score indicates a superior system, and even-number items are negative questions, where a lower score indicates a superior system. Table 2 outlines the SUS assessment items and contents.

The score of each item was converted to a scale of 100. For odd-number items, the total score was subtracted by 5 to obtain (X), and for even-number items, the total score was subtracted from 25 to obtain (Y).

Then, the sum of the two values (X + Y) was multiplied by 2.5 to obtain the overall SUS score [15]. Finally, the overall SUS score was classified according to the grade scale, an adjective rating, acceptance level, recommendation, and as shown in Table 3 [16,17]. 

### 2.4. Satisfaction Evaluation 

User satisfaction was evaluated using 5 assessment items on the overall system satisfaction, architecture and understandability, degree of interest, recommendation, and continuous use intention. Each item was rated on a 5-point Likert scale as in the SUS items. An item score was obtained by subtracting 1 from the point gained and multiplying the resulting value by 25, thus converting to a scale of 100 (range: 0–100). 

### 2.5. Data Analysis

Data analysis was performed on the sociodemographic variables (gender, age, occupation, and career) to calculate frequency and percentage using Microsoft Office Excel 2007 software. Overall mean SUS and user satisfaction scores and individual item scores were compared, and SUS score classification was compared and analyzed.

## 3. Results

### 3.1. Participants Sociodemographic Characteristics

For usability evaluation, 43 physical disability-related healthcare professionals participated in the study. Their sociodemographic characteristics were analyzed as follows: gender distribution was 38 men (88.4%) and five women (11.6%); the most frequent age group was the 40s (n = 25, 58.1%), followed by the 30s (n = 5, 11.6%); occupation distribution was 26 academics (60.5%), nine public health and welfare professionals (20.9%), and eight industrial practitioners (18.6%); the most frequent career length was 10–19 year (n = 22, 51.2%) (Table 4).

### 3.2. Usability Scale Rating 

**Figure 3:**
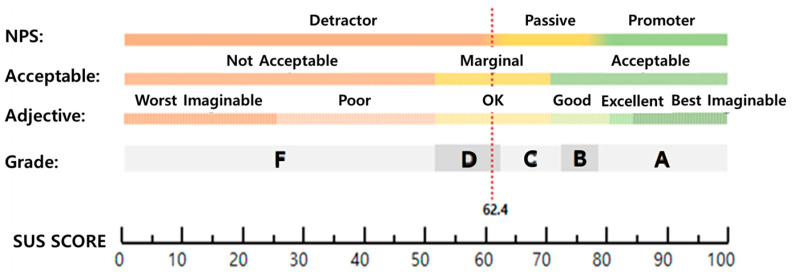
SUS scores and standards.

**Figure 4:**
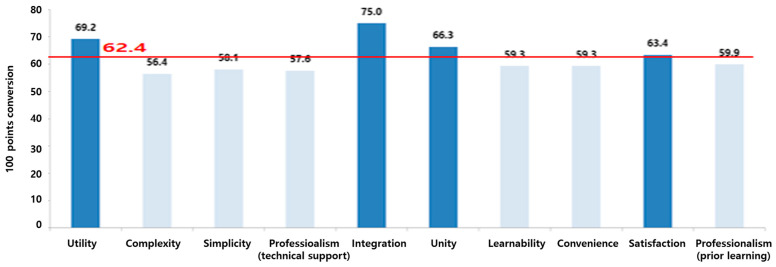
SUS item scores (Converted to a scale of 100).

The overall SUS mean score was 62.4, which corresponds to grade “D” on the grade scale in the SUS score classification (Table 3, Figure 3). Items with lower values than the overall mean SUS score were identified as complexity (Q2), convenience (Q8), simplicity (Q3), professionalism (technician support, prior learning) (Q4, Q10), and learnability (Q7) (Table 5, Figure 4).

### 3.3. User Satisfaction Evaluation

**Figure 5:**
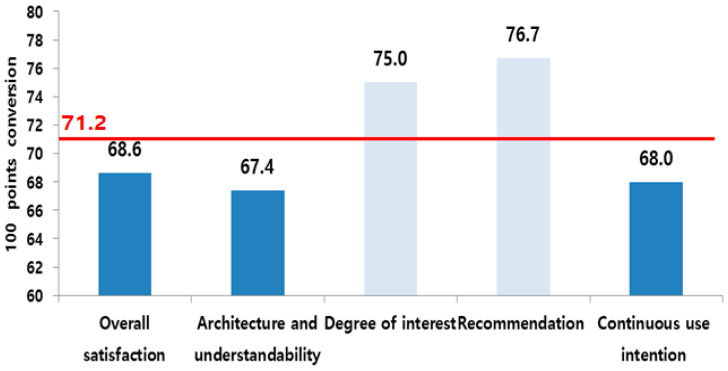
Satisfaction item scores (converted to a scale of 100).

The overall satisfaction mean score was 71.2, with the item’s overall satisfaction, architecture and understandability, and continuous use intention marking lower scores than the overall mean value (Table 6, Figure 5).

## 4. Discussions

In this study, we conducted a formative usability evaluation of a three-way digital healthcare system, which was developed to build a health and safety net for people with disabilities, with the participation of healthcare professionals with the aim of deriving the directions of system improvement and providing basic data for further system enhancement.

SUS scores are divided into rating categories of best imaginable, excellent, good, fair, poor, and worst imaginable, and a SUS score lower than 69 (the 50th percentile) indicates problems requiring investigation or solution [15,18]. In this study, the overall mean SUS score was 62.4, which corresponds to SUS Grade “D,” adjective rating of “fair,” and an acceptance level of “nearly acceptable” [16,17] (Figure 3).

Formative usability evaluation has resulted in low usability during the evaluation since the expert’s objective identification and analysis were utilized rather than the users’ point of view.

Among the 10 SUS items, four items scored above average (≥62.4): utility (69.2), integration (75.0), unity (66.3), and satisfaction (63.4), and six items scored below average (<62.4): complexity (56.4), simplicity (58.1), professionalism (technician support) (57.6), learnability (59.3), convenience (59.3), and professionalism (prior learning) (59.9). Thus, it was confirmed that the overall system architecture needs to be improved, focusing on complexity, simplicity, professionalism, learnability, and convenience. In particular, the fact that simplicity and complexity marked the lowest scores highlights the urgency of improving the system towards simplicity and convenience (Figure 4). To improve simplicity and complexity, the UI needs to be changed to be simple and intuitive.

The mean user satisfaction score was calculated at 71.2. Among the satisfaction items, the overall satisfaction score was 68.6, which is higher than system architecture and understandability (67.4) and continuous use intention (68.0) and lower than the degree of interest (75.0) and recommendation (76.7) (Figure 5). These results coincide with the low scores in SUS complexity and simplicity and also highlight the need to enhance the system’s simplicity.

Factors affecting the usability and durability of healthcare-related wearable devices include various technological factors, interactive factors, and psychological factors such as motivation and sense of achievement [19]. Among them, simplicity was found to be positively associated with the continuous use of information systems from the utility aspect [20], and ease of use was identified as a determinant factor for utility and attitude of use, which was positively associated with continuous use intention [21].

In this study, we meticulously examined all system problems and improvement needs and sought methods to improve system simplicity for ease of use and efficient user environments. By building an online health and safety net for people with disabilities, it is possible to help them live healthy lives and respond to emergency situations in this COVID-19 era. It is expected that a self-directed health management environment can be created by enabling people with disabilities to report patient-generated health data (PGHD) for themselves and caregivers and to implement the monitoring system. In this context, it is also hoped that standardized health management guidelines for people with disabilities will be provided for professionals in related health and welfare facilities, as well as remote healthcare delivery to people with disabilities in crisis situations such as COVID-19 in linkage with primary care physicians of people with disabilities, community health centers, and local healthcare centers for people with disabilities. By implementing the improved tripartite health information sharing system, various effects are expected, such as the health and safety of people with disabilities and their caregivers, promotion of healthy living, mutual encouragement to lead a healthy life, enhancement of quality of life, communication, and participation.

At the current stage of app development, this utility evaluation study was conducted first with healthcare professionals. To develop a high-performing app, it is first necessary to improve an existing app, then to perform usability evaluation with the participation of people with disabilities, who are target users, and their caregivers, and finally to further improve the app based on the results of usability evaluation.

A successfully upgraded app can be an effective healthcare service model for the comprehensive health management of not only people with disabilities but also their caregivers. In addition, it would be a highly significant system solution to integrate the tripartite sharing-based digital healthcare system into the envisaged local community health management project system for people with disabilities in that it can become a high-usability integrated health and welfare service model for people with disabilities.

## 5. Conclusions

This study analyzed comprehensive improvement directions for system architecture improvement centering on complexity (Q2), simplicity (Q3), professionalism (technician support, prior learning) (Q4, Q10), learnability (Q7), and convenience (Q8) with the aim of enhancing the usability of a newly developed digital healthcare system. It is necessary to make continuous efforts to improve system satisfaction and continuous use intention by deriving concrete system improvement needs and addressing those needs through system enhancement. In addition, usability evaluations of improving digital healthcare systems are required by end users, such as people with disabilities and their caregivers. It is expected that such research outcomes will contribute to developing an effective healthcare management service model that addresses all problems facing people with disabilities and their caregivers.

## Figures and Tables

**Table 1:** Architecture of a Three-Way Digital Healthcare System for the People with Disabilities and Their Caregivers.

Depth 1	Depth 2	Depth 3
1. My health at a glance ((Step count, sleep duration, weight, BMI) *, blood glucose, blood pressure, ten-mission checklist, favorites)		
2. My profile	Account management	Basic information
		Information sharing selection * ( **P** **eople with disabilities or their caregiver’s)**
	My health checks (People with disabilities, caregiver)	Evaluation date *
		Activities of daily living evaluation (MBI)
		Quality of life evaluation (EQ-5D)
		Motor function evaluation (MAS)
		Depression (PHQ-9) *
		Anxiety (GAD-7) *
		Cognitive function index
		Health behavior * **(Drinking, smoking, blood pressure, blood glucose level, height,** **weight, BMI, pain, daily calorie intake, calorie intake check against the** **recommended level, sodium content, sugar content)**
		Medication (compliance, drug type)
		Consultation log (required items check)
3. My health	My health coaching	Exercise
		Nutrition
		Mind *
	My daily health records	Mission setting and notice (**(Drinking, smoking, nutrition, exercise,** medication, mental health, smart band, smart scale) *, smart blood pressure monitor, smart blood glucose monitor)
		Life log ((Smart band, smart scale) *, smart blood pressure monitor, smart blood glucose monitor)
		Self-report log * (Nutrition log, medication log, other required items)
	My routine health checks	Mission completion check*
		Life log records check *
		Self-report outcome check *
4. My wellbeing assistant	Health information	Disability-related disease management *
		Chronic disease management *
	Safety and welfare information	Infectious disease crisis response *
		Welfare and policy information at a glance *
		Assistive and exercise equipment*
		Disability-friendly healthcare and welfare facilities *
		Mobility and transportation*
		Guide for emergency response*
		Improving disability-related information/awareness *
		Sport facilities*
5. Health together (Comparison of records)	My trophy	**Frequency of mission completion (** **C** aregiver + disabled person) *
	Weekly health life evaluation outcome (Weekly Report)	**Visualized “My health at a glance” data of people with disabilities** (Caregiver’s opinion) *
		Visualized “My health at a glance” data of Caregiver (People with disabilities’ opinion)
6. Community	People with disabilities community	
	Caregiver community *	
	Questions and answers *	

Items in bold and with * are caregiver-enabled features, MBI: Modified Bathel Index, EQ-5D: Euro Qol-5D, MAS: Motor Assessment Scale, PHQ-9: Patient Health Questionnaire-9, GAD-7: General Anxiety Disorder-7, BMI: Body Mass Index.

**Table 2:** System Usability Scale (SUS) questionnaire.

Items	Statement
1. Utility	I think that I would like to use this system frequently.
2. Complexity	I found the system unnecessarily complex.
3. Simplicity	I thought the system was easy to use.
4. Professionalism (Technician support)	I think that I would need the support of a technical person to be able to use this system.
5. Integration	I found the various functions in the system were well integrated.
6. Unity	I thought there was too much inconsistency in this system.
7. Learnability	I would imagine that most people would learn to use this system very quickly.
8. Convenience	I found the system very cumbersome to use.
9. Satisfaction	I felt very confident using the system.
10. Professionalism (Prior learning)	I needed to learn a lot of things before I could get going with this system.

**Table 3:** SUS score classification.

Grade Scale	Range	Percentile Range	Adjective Rating	Acceptance Level	Recommendation
A+	84.1–100	96–100	Best imaginable	Acceptable	Recommendable
A	80.8–84.0	90–95	Excellent		
A−	78.9–80.7	85–89			
B+	77.2–78.8	80–84			Neutral
B	74.1–77.1	70–79			
B−	72.6–74.0	65–69			
C+	71.1–72.5	60–64	Good		
C	65.0–71.0	41–59		Nearly acceptable	
C−	62.7–64.9	35–40			
D	51.7–62.6	15–34	Fair		Unrecommendable

**Table 4:** Participants sociodemographic characteristics (Unit: n, %).

Category		Frequency (%)
Gender	M	38 (88.4%)
	F	5 (11.6%)
Age Group	30 s	5 (11.6%)
	40 s	25 (58.1%)
	50 s	12 (27.9%)
	60 s	1 (2.3%)
Occupation	Academia	26 (60.5%)
	Public health and welfare	9 (20.9%)
	Industry	8 (18.6%)
Career	<10 years	7 (16.3%)
	10–19 years	22 (51.2%)
	20–29 years	10 (23.3%)
	30–39 years	3 (7.0%)
	≥40 years	1 (2.3%)

**Table 5:** SUS evaluation results. (N = 43, Unit: point).

Item	Min	Max	M ± SD	Scaled M ± SD
Total	-	-	3.10 ± 0.88	62.4 ± 15.7
Utility	2.00	5.00	3.77 ± 0.68	69.2 ± 17.1
Complexity	1.00	4.00	2.74 ± 0.88	56.4 ± 21.9
Simplicity	1.00	5.00	3.33 ± 0.81	58.1 ± 20.2
Professionalism (technician support)	1.00	5.00	2.70 ± 1.17	57.6 ± 29.1
Integration	2.00	5.00	4.00 ± 0.69	75.0 ± 17.3
Unity	1.00	4.00	2.35 ± 0.69	66.3 ± 17.2
Learnability	1.00	5.00	3.37 ± 1.05	59.3 ± 26.2
Convenience	1.00	4.00	2.63 ± 1.02	59.3 ± 25.6
Satisfaction	2.00	5.00	3.53 ± 0.77	63.4 ± 19.2
Professionalism (prior learning)	1.00	5.00	2.60 ± 1.00	59.9 ± 25.1

M: Mean, SD: Standard Deviation.

**Table 6:** Satisfaction evaluation results (N = 43, Unit: point).

Item	Min	Max	M ± SD	Scaled M ± SD
Total	-	-	3.85 ± 0.73	71.2 ± 20.7
Overall satisfaction	2.00	5.00	3.74 ± 0.76	68.6 ± 19.0
Architecture and understandability	2.00	5.00	3.70 ± 0.77	67.4 ± 19.3
Degree of interest	3.00	5.00	4.00 ± 0.69	75.0 ± 17.3
Recommendation	3.00	5.00	4.07 ± 0.59	76.7 ± 14.8
Continuous use intention	2.00	5.00	3.72 ± 0.85	68.0 ± 21.4

M: Mean, SD: Standard Deviation.

## Data Availability

The data used and/or analyzed during the current study are available from the corresponding author on request.
